# Supplementary material for: A low-threshold intervention to increase physical activity and reduce physical inactivity in a group of healthy elderly people in Germany: Results of the randomized controlled MOVING study
Source: PLoS One. 2021 Sep 16;16(9):e0257326. doi: 10.1371/journal.pone.0257326 (PMC8445413; doi:10.1371/journal.pone.0257326)
Supplement: S1 Table — Notes: n number of subjects, SD standard deviation. (PDF) [file pone.0257326.s001.pdf]

| | Control group | | Intervention group | | Both groups | |
|---|---|---|---|---|---|---|
| | n | Mean (SD) or n (%) | n | Mean (SD) or n (%) | n | Mean (SD) or n (%) |
| ***Characteristics*** | | | | | | |
| *Sex (women)* | *81* | *47* (58.0%) | *85* | 50 (58.8%) | *166* | 97 (58.4%) |
| *Age (yr)* | *81* | 71.2 (SD 5.0) | *85* | 70.4 (SD 4.6) | *166* | 70.8 (SD 4.8) |
| *Number of participants currently living in a partnership (yes)** | *77* | 47 (61.0%) | 83 | 67 (80.7%) | *160* | 114 (71.3%) |
| *Education (yr)* | *74* | | 82 | | *156* | |
| *< 10 years* | | 14 (18.9%) | | 18 (22.0%) | | 32 (20.5%) |
| *= 10 years* | | 25 (33.8%) | | 17 (20.7%) | | 42 (26.9%) |
| *> 10 years* | | 30 (40.5%) | | 45 (54.9%) | | 75 (48.1%) |
| *Other* | | 5 (6.8%) | | 2 (2.4%) | | 7 (4.5%) |
| *Body mass index (kg/m²)* | *81* | | 85 | | *166* | |
| *< 25* | | 22 (27.2%) | | 23 (27.1%) | | 45 (27.1%) |
| *≥ 25 and < 30* | | 33 (40.7%) | | 30 (35.3%) | | 63 (38.0%) |
| *≥ 30* | | 26 (32.1%) | | 32 (37.6%) | | 58 (34.9%) |
| *Waist circumference (cm)* | *81* | 96.7 (SD 13.1) | 85 | 94.8 (SD 14.6) | *166* | 95.8 (SD 13.9) |
| *Hip circumference (cm)* | *81* | 104.1 (SD 11.8) | 85 | 102.7 (SD 10.4) | *166* | 103.4 (SD 11.1) |
| *Blood pressure (sys/dia mmHg) (Pulse /min)* | *81* | 133/74 (70) | 84 | 132/72 (73) | *165* | 133/73 (71) |
| *Wearing time of the accelerometer* | | | | | | |
| *Baseline* | *81* | 5,922.7 (SD 926.8) | 85 | 5,945.7 (SD 637.2) | *166* | 5,934.5 (SD 789.5) |
| *3-month follow-up* | *81* | 5,833.6 (SD 839.1) | 84 | 5,826.4 (SD 683.9) | *165* | 5,829.9 (SD 761.7) |
| *6-month follow-up* | *79* | 5,636.4 (SD 719.7) | 82 | 5,840.7 (SD 719.0) | *161* | 5,740.5 (SD 724.4) |
