# Supplementary material for: A low-threshold intervention to increase physical activity and reduce physical inactivity in a group of healthy elderly people in Germany: Results of the randomized controlled MOVING study
Source: PLoS One. 2021 Sep 16;16(9):e0257326. doi: 10.1371/journal.pone.0257326 (PMC8445413; doi:10.1371/journal.pone.0257326)
Supplement: S2 Table — Notes: n number of subjects, CI 95% confidence interval, M mean. (PDF) [file pone.0257326.s002.pdf]

Study phase	Study Group	Physical inactivity [min.] M (CI 95%)	Sedentary breaks M (CI 95%)	Light PA [min.] M (CI 95%)	Moderate PA [min.] M (CI 95%)	Vigorous PA [min.] M (CI 95%)	Overall PA [min.] M (CI 95%)	Steps Counts M (CI 95%)
**Baseline**	Intervention group (n=85)	3482.0 (3336.1-3627.8)	97.6 (92.7-102.4)	2246.4 (2139.9-2352.8)	213.6 (180.6-246.6)	3.8 (0.6-7.0)	2463.8 (2342.8-2584.8)	99,278.0 (92,889.8-105,666.2)
	Control group (n=81)	3438.5 (3234.3-3642.7)	95.9 (89.3-101.9)	2276.1 (2154.4-2397.8)	201.1 (166.5-235.6)	6.6 (1.2-14.3)	2484.1 (2357.2-2611.1)	97,763.2 (90,840.4-104,685.9)
	Total (n=166)	3460.8 (3337.5-3584.0)	96.8 (93.0-100.6)	2260.9 (2181.1-2340.7)	207.5 (183.8-231.1)	5.1 (1.1-9.2)	2473.7 (2387.0-2560.4)	98,538.8 (93,884.7-103,193.0)
**3-months follow-up**	Intervention group (n=84)	3351.1 (3204.8-3497.3)	92.4 (87.6-97.0)	2292.3 (2174.7-2409.8)	180.3 (148.3-212.3)	2.7 (0.2-5.6)	2475.3 (2348.7-2602.0)	99,554.3 (93,268.3-105,840.2)
	Control group (n=81)	3318.8 (3140.0-3497.7)	93.6 (87.8-99.4)	2327.5 (2188.9-2466.2)	181.5 (151.0-212.0)	5.3 (1.0-9.7)	2514.8 (2369.9-2659.6)	97,567.4 (90,803.8-104,330.9)
	Total (n=165)	3335.3 (3221.3-3449.2)	93.0 (89.4-96.6)	2309.6 (2219.9-2399.3)	180.9 (159.0-202.8)	4.0 (1.4-6.6)	2494.7 (2399.7-2589.7)	98,578.9 (94,013.7-103,144.1)
**6-months follow-up**	Intervention group (n=83)	3354.8 (3210.0-3499.6)	92.5 (88.1-96.9)	2300.4 (2180.1-2420.8)	184.9 (154.2-215.6)	3.3 (0.7-5.8)	2488.8 (2358.9-2618.2)	99,613.6 (93,526.0-105,701.2)
	Control group (n=79)	3228.2 (3087.0-3369.4)	90.2 (85.6-94.7)	2216.9 (2077.8-2356.0)	187.7 (150.3-225.1)	3.5 (1.6-8.5)	2408.2 (2263.0-2553.4)	94,301.4 (87,290.4-101,312.5)
	Total (n=162)	3293.1 (3192.5-3393.7)	91.4 (88.2-94.5)	2259.7 (2168.8-2350.6)	186.3 (162.5-210.1)	3.4 (0.6-6.2)	2449.5 (2353.2-2545.8)	97,023.1 (92,426.6-101,619.7)
