# Supplementary material for: A low-threshold intervention to increase physical activity and reduce physical inactivity in a group of healthy elderly people in Germany: Results of the randomized controlled MOVING study
Source: PLoS One. 2021 Sep 16;16(9):e0257326. doi: 10.1371/journal.pone.0257326 (PMC8445413; doi:10.1371/journal.pone.0257326)
Supplement: S3 Table — (PDF) [file pone.0257326.s003.pdf]

WHO recommendation	Study group	Baseline (n=166, IG=85, CG=81)	3-month follow-up (n=165, IG=84, CG=81)	6-month follow-up (n=162, IG=83, CG=79)
**moderate PA ≥150 min. per week or vigorous PA ≥75 min. per week**	Intervention group	49 (57.6%)	43 (51.2%)	41 (49.4%)
	Control group	48 (59.3%)	39 (48.1%)	38 (48.1%)
	Total	97 (58.4%)	82 (49.7%)	79 (48.8%)
