# Supplementary material for: A low-threshold intervention to increase physical activity and reduce physical inactivity in a group of healthy elderly people in Germany: Results of the randomized controlled MOVING study
Source: PLoS One. 2021 Sep 16;16(9):e0257326. doi: 10.1371/journal.pone.0257326 (PMC8445413; doi:10.1371/journal.pone.0257326)
Supplement: Attachment — Submitted filename: R-1.docx [file pone.0257326.s005.docx]

**Recommendations:**

**Abstract**

1. The acronyms generate confusion. It is suggested that PA be used to define physical activity and PI to use physical inactivity.

**Introduction**

1. I suggest that the introduction be restructured as follows:

-Definition of physical inactivity (PI)

*Types of physical inactivity (high, low …)

*Prevalence in the age group

*Effects on physical or mental health.

-Definition of physical activity (PA)

*Types of physical activity (light, regular, moderate, vigorous) (structured or incidental)

*Prevalence in your age group

*Effects on physical or mental health.

* WHO recommendations

-Interaction between PI and PA (Are there subjects with high PA and high PI ?)

- The interventions should be separated by type of PA or PI and the.

**Methods**

1. Line 163. Define each test applied and describe applied and describe what each of them evaluates (e.g., IPAQ??)
2. Line 182. Indicate which recommendations were given to the subjects based on their age.

**Data analysis**

1. Given that you are assessing light, moderate and vigorous PA, you should use these terms since the introduction section.
2. Line 189 Explains why it decided to exclude these participants under these criteria.
3. I suppose that the use of t-tests for statistical analysis may require some statistical correction.
4. Given that the sample has a broad age range, I suggest performing analyses separating groups by sex or age, for example, participants between 65 and 75 years old. Maybe you will find significant differences.
5. Did you apply the tests before and after the intervention? e.g., blood pressure, measurement of body weight, waist, and hip circumference. Maybe you have a weight status or fitness effects that you are not assessing.

**Results**

1. I suggest to report the differences between the values before and after interventions for the treated and controls group. After that, you inform the comparisons between treated the control group.

**Discussion**

1. In the discussion section, you discuss and conclude about other issues than your data, e.g., line 292, *our data support that the use of an accelerometer is feasible and practical in this age group.* Given that your intervention does not affect PA or PI, the accelerometer was irrelevant for this age group; thus, your conclusion is wrong.

**Minor**

-Line 59 replace “high levels of physical inactivity” with “high levels of PI.”

-Line 64 use the acronym PI, replace “physical inactivity” with “PI.”

-Lines 72 and 82, you defined the MVPA twice. I suggest replacing “MVPA” with “moderate or vigorous PA.”

-Lines 58, 71, and 84 replace physical activity with PA.
