# Supplementary material for: A low-threshold intervention to increase physical activity and reduce physical inactivity in a group of healthy elderly people in Germany: Results of the randomized controlled MOVING study
Source: PLoS One. 2021 Sep 16;16(9):e0257326. doi: 10.1371/journal.pone.0257326 (PMC8445413; doi:10.1371/journal.pone.0257326)
Supplement: Attachment — Submitted filename: Response to Reviewers.docx [file pone.0257326.s006.docx]

**PONE-D-21-04633**

A low-threshold intervention to increase physical activity and reduce physical inactivity in a group of healthy elderly people in Germany: Results of the randomized controlled MOVING study

PLOS ONE

**Response to Reviewers**

*Thank you for coordinating the review process and for your constructive comments to the manuscript. In our opinion, the comments help to increase the quality of the manuscript, especially the part of the statistical analyses. We have revised the manuscript again and addressed all reviewer’s comments from the minor revision.*

**Reviewer #1**

Comment: I am happy with the authors' responses. Some minor comments and perhaps advice for future work:

1) try to use a regression model over ANOVA. it is more flexible, easier to interpret and allows for controlling for numerous covariates.

*Response: Thank you for this comment to the statistical analysis. In the revised version of the manuscript, we provide a Two-Way Mixed ANOVA with repeated measures that is in line with reviewer 2 from the major revision. For further analysis, we will take into account the advice from reviewer 1 and consider using a regression model.*

Comment: baseline characteristics appear similar across the two groups. report as mean (95% CI) rather than M (95% CI) in the respective table. You could include comparisons at baseline between the two groups, although I don't think anything is significantly different.

*Response: At baseline, we checked all variables regarding significant group differences. Here, we detected one systematic difference in the variable “partnership” between intervention and control group. Therefore, we added the variable “partnership” as a covariate in the Two-Way Mixed ANOVA to mitigate the effect. All other variables in table 1 were not significantly different. In the revised version of the manuscript, we added the following sentence: “For baseline data, we analyzed the equality of variables between both study groups.” (please see lines: 210 – 211).*

*We changed the report of table 2* that is consistent with Reviewer 1 recommendations (Mean (95% CI - *please see green color in table 2).*

Comment: sensitivity rather than sensitive analyses

*Response: Thank you for this comment. In the revised version of the manuscript, we addressed the comment.*

Comment: in the absence of a main effect we don't tend to examine for interactions

*Response: Thank you for this comment. We followed your recommendation and adapted the section with very few changes without reporting the details about the interaction effect. Now, we summarize the details about the interaction effect in one sentence: “P-values were ranging between 0.317 to 0.546.”. (Please see line: 260).*

**Reviewer #3**

Comment: The authors should provide the meaning of each line color in figure 2, and correct the usage of parentheses and brackets in the results section.

*Response: Thank you for this comment. The blue line represents the intervention group and the red line represents participants in the control group. We also added the meaning of each line in the manuscript (line 247). In the revised version of the manuscript, we have addressed Reviewer 3 comments and corrected the usage of parentheses and brackets in the results section (please see changes in green color and review mode in the manuscript).*
