# Supplementary material for: A low-threshold intervention to increase physical activity and reduce physical inactivity in a group of healthy elderly people in Germany: Results of the randomized controlled MOVING study
Source: PLoS One. 2021 Sep 16;16(9):e0257326. doi: 10.1371/journal.pone.0257326 (PMC8445413; doi:10.1371/journal.pone.0257326)
Supplement: Attachment — Submitted filename: Response to Reviewers.docx [file pone.0257326.s007.docx]

**PONE-D-21-04633**

A low-threshold intervention to increase physical activity and reduce physical inactivity in a group of healthy elderly people in Germany: Results of the randomized controlled MOVING study

PLOS ONE

**Response to Reviewers**

*Thank you for coordinating the review process and for your constructive comments to the manuscript. In our opinion, the comments help to increase the quality of the manuscript, especially the part of the statistical analyses. We have revised the manuscript again and addressed all reviewer’s comments from the minor revision.*

**Reviewer #1**

Comment: I consider that the article could be accepted after it satisfies the following requests:

1. The authors should provide the meaning of each line color in figure 2.

*Response: The blue line represents the intervention group and the red line represents participants in the control group. We also added the meaning of each line in the manuscript (please see lines 247, 249 and 608/609 in “manuscript_with_track_changes”).*

Comment: 2. The authors should correct the usage of parentheses and brackets in the results section.

*Response: In the revised version of the manuscript, we have addressed Reviewer 3 comments and corrected the usage of parentheses and brackets in the results section (please see changes in green color and review mode in the manuscript).*

Comment: 3. Please report as mean (95% CI) rather than M (95% CI) in the respective table.

*Response: In the revised version of the manuscript, we changed the report of Mean (95% CI)* that is consistent with Reviewer 1 recommendations. *Please see Table 2 in the manuscript.*

Comment: 4. Please include comparisons at baseline between the two groups.

*Response: At baseline, we checked all variables regarding significant group differences. Here, we detected one systematic difference in the variable “partnership” between intervention and control group. Therefore, we added the variable “partnership” as a covariate in the Two-Way Mixed ANOVA to mitigate the effect. Other variables in table 1 were not significantly different. In the revised version of the manuscript, we added the following sentence: “For baseline data, we analyzed the equality of variables between both study groups.” (please see lines: 210 – 211).*

Comment: 5. Please, modify your wording: "sensitivity" rather than "sensitive analyses"

*Response: Thank you for this comment. In the revised version of the manuscript, we addressed the comment.*
